# Comprehensive Evaluation of OS Starch–Oleic Acid Mixtures: From Functional Properties to Their Application in Films with Improved Water Resistance

**DOI:** 10.3390/molecules30224411

**Published:** 2025-11-14

**Authors:** Karolina Królikowska, Paulina Pająk, Sławomir Pietrzyk, Karolina Czaplak, Katarzyna Strządała

**Affiliations:** Department of Food Analysis and Evaluation of Food Quality, University of Agriculture, Balicka 122 Str., 30-149 Krakow, Poland; paulina.pajak@urk.edu.pl (P.P.); slawomir.pietrzyk@urk.edu.pl (S.P.);

**Keywords:** octenyl succinate starch, oleic acid, starch–lipid interactions, edible films, starch modification

## Abstract

This study investigated the effects of octenyl succinate (OS) starches mixed with oleic acid on functional properties and potential use in edible films. Potato starches esterified with 1%, 3%, 5%, or 7% of octenyl succinic anhydride (OSA) were mixed with oleic acid. Degree of substitution (DS), hydrodynamic volume, and lipid content were measured to evaluate effectiveness of modification. Blank sample and modified starches were analyzed for water binding capacity, solubility, characteristic of gelatinization, pasting properties, and surface/interfacial tensions. Edible films were prepared from the obtained starches and tested for water vapor permeability, water binding capacity, and solubility. The complexation index increased linearly with DS. Oleic acid reduced water binding capacity and solubility, particularly at 80 °C, altered thermodynamic characteristic of gelatinization, decreased viscosities of OS starch pastes, and increased pasting temperatures by up to 20%. It also enhanced the surface tension lowering effect of OS starch and reduced water vapor permeability in films, especially at higher DS. Films from starch–oleic acid mixtures exhibited lower water binding capacity and solubility, notably in 5% and 7% OSA modified starch. Results show that oleic acid addition to OS starch markedly affect functional properties of starch, highlighting its potential for use in edible film applications.

## 1. Introduction

Octenyl succinate starch is a chemically modified starch produced during the esterification reaction between starch hydroxyl groups and octenyl succinic anhydride. The introduction of hydrophobic groups in the form of OS groups into starch chains result in an amphiphilic character of the entire molecule. Due to their amphiphilic character, OS starches are surface-active and can adsorb at interfaces, stabilizing dispersions [1,2]. Due to these unique properties, OS starches have a wide variety of applications, particularly in emulsification, encapsulation, and in the production of films, coatings, and gels [3,4,5]. Octenyl succinylation of starch has been approved for use as a food additive in the European Union [6].

The amphiphilic nature of OS starch enables interactions with other macromolecules. In recent years, there have been some reports on interactions of OS starch with various food ingredients, both in solution and emulsion systems. Magnusson & Nilsson [7] studied the interactions between OS starches and α-β-livetin the water-soluble fraction of egg yolk. The authors showed that the interaction between the studied compounds was associative in nature, leading to the formation of livetin-OS-starch complexes. These complexes arise from hydrophobic and electrostatic interactions between starch and livetin. It has been also demonstrated that OSA modified starch can interact with proteins including gelatin and casein [8].

Besides proteins, lipids are another type of food components that interact with starch. Examples of such interactions include inclusion complexes formed between the left-handed amylose helix of starch and fatty acids or monoglycerides. In these structures, the hydrocarbon chain of the lipid resides in a central hydrophobic cavity of the amylose helix. The obtained starch derivatives show significantly different properties and potential functionalities compared to native starches. So far, extensive studies on starch–lipid complexes have been conducted in terms of their formation mechanism [9,10], structure [11,12], functionality [13,14], and enzymatic hydrolysis susceptibility [15]. One of the main application fields of amylose–lipid complexes is in microencapsulation processes. It has been demonstrated that the introduction of conjugated linoleic acid into the amylose helix improve the oxidative stability of this fatty acid [16]. Importantly, most previous studies focused on well-defined complexes, typically formed after solubilization of starch and lipids under heating or in organic solvents. In contrast, the present work investigates mixtures in which lipids are incorporated into starch without prior solubilization, thereby preserving the granular integrity. In the method of starch–lipid mixture preparation, the selected lipid is firstly dissolved in ethanol or another organic solvent, then added to starch, and the whole mixture is stirred to complete evaporation of the solvent. Obtained starch derivatives retain granule integrity and exhibit thickening properties. In the case of starch–fatty acid mixtures, inclusion complexes are formed during starch gelatinization [17,18]. So far, there have been no reports in the literature describing interactions between octenyl succinate starch and lipids in relation to their mixtures. However, the emulsifying properties of OSA-modified starch may influence its interaction with lipids. It was proven that the steric effects and electrostatic repulsion between molecules imposed by OS groups counteract hydrogen bonding between starch chains, thereby affecting the structural characteristics and potentially the susceptibility of the starch to interact with lipids [19].

Oleic acid is a mono-unsaturated omega-9 fatty acid. Enrichment of foods that are naturally sources of saturated fatty acids with oleic acid has been reported to reduce cardiovascular risk by reducing blood lipids, mainly cholesterol. As intakes of saturated fats in many countries exceed that of the recommended levels, an increase in the intake of oleic acid will be beneficial for public health [20,21]. In our previous study, it was reported that mixtures of cassava and wheat starches with oleic acid showed higher complexing index values compared to their palmitic or stearic acid counterparts. The presence of oleic acid limited water binding capacity and solubility, increased final viscosity, enhanced rheological stability, and contributed to higher resistant starch content [22]. The amphiphilic and emulsifying properties of OS starch suggest that its mixtures with oleic acid may exhibit unique structural and functional features. The use of OS starch as a carrier of oleic acid in the diet represents a potentially innovative approach to producing health-promoting food that positively impacts the nutritional status of the population while maintaining the technological functionalities of the starch.

Beyond food applications, starch and lipid components have attracted increasing attention in material science, particularly for the production of biodegradable films. So far, it has been reported that films prepared from OS starch exhibit better water barrier properties compared to films prepared from other modified starches [23]. Our recent studies have also shown that OS starch films modified with bioactive ethanolic extracts of honey bee products exhibit enhanced functional properties compared to native starch films [24,25]. These findings suggest that OS starch can act as a versatile film-forming material, able to improve mechanical, barrier, and functional properties when combined with lipophilic or bioactive compounds. The addition of lipids to the coating material can further enhance the mechanical strength and barrier properties of the final product [26]. Moreover, the use of OS starch as a film-forming material may facilitate the uniform dispersion of lipophilic oleic acid throughout the film matrix. Thus, OS starch–oleic acid mixtures represent a promising approach for developing films with improved water barrier performance and homogeneous lipid distribution. However, films prepared from OS starch–lipid mixtures have not yet been investigated.

The behavior and functional characteristics of starch–lipid mixtures, particularly those involving octenyl succinate–modified starch, have not been extensively studied. Therefore, it is of high scientific interest to explore how OS starch interacts with oleic acid in such mixtures and to systematically evaluate the resulting structural, thermal, and functional properties, including the formation of starch-based films, with enhanced water resistance. We hypothesize that the degree of OS substitution affects the extent of starch–oleic acid interactions, influencing complex formation, pasting behavior, and water barrier properties of the films. This study provides novel insights into the structure–function relationships of OS starch–lipid systems and offers a comprehensive evaluation of their potential for designing starch-based materials with controlled water resistance, which has not been previously reported.

## 2. Results and Discussion

### 2.1. Evaluation of Oleic Acid-Octenyl Succinate Starch Mixture Properties

#### 2.1.1. Effectiveness of Modification Procedures

As presented in Table 1, the DS of OS starches increased with an increase in the concentration of octenyl succinic anhydride added to the starch, which was in agreement with previous studies [27,28]. The content of octenyl succinate groups in the starch was lower than 3% (calculated per dry mass of starch (d.m.)), complying with the requirements regarding starch approval for use in the food industry [6].

**Figure 1:**
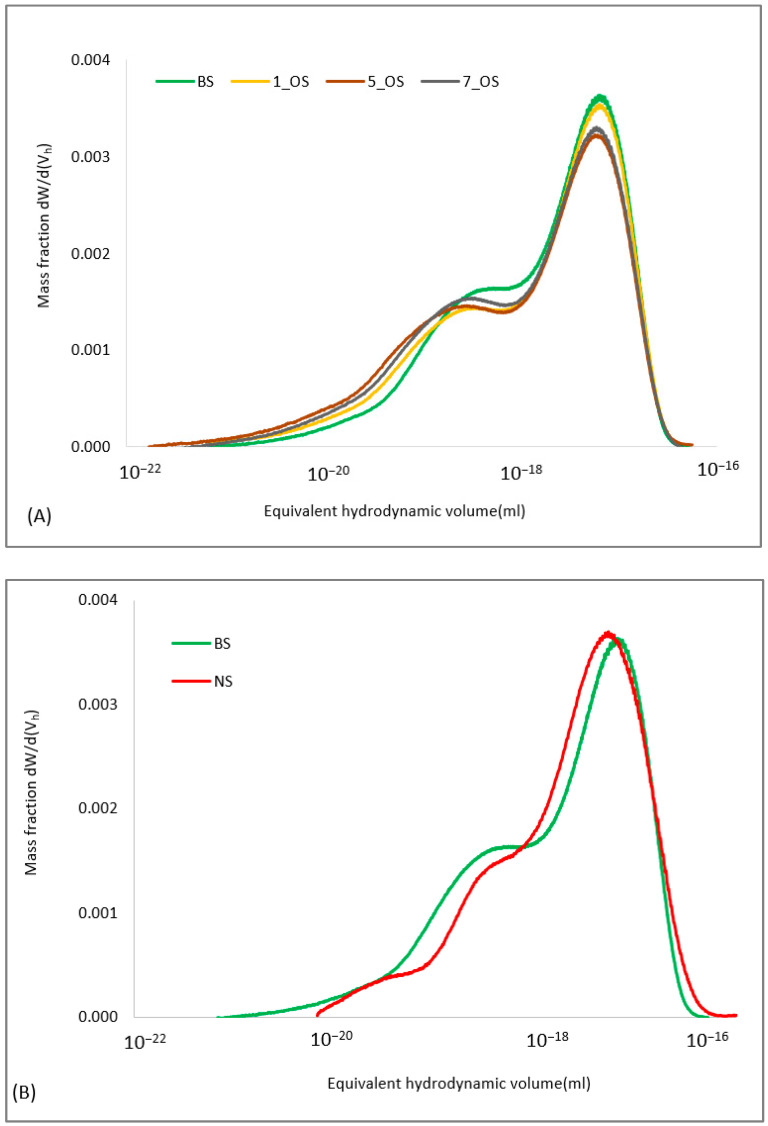
Equivalent hydrodynamic volume distributions of (**A**) blank sample, octenyl succinate starches, and their mixtures with oleic acid, and (**B**) native starch and blank sample. NS: native starch; BS: blank sample; 1_OS: octenyl succinate starch modified with 1% OSA; 3_OS: octenyl succinate starch modified with 3% OSA; 5_OS: octenyl succinate starch modified with 5% OSA; 7_OS: octenyl succinate starch modified with 7% OSA; BS+OA, 1_OS+OA, 3_OS+OA, 5_OS+OA, 7_OS+OA: corresponding starch samples mixed with oleic acid.

The OS starch was subjected to an analysis of the hydrodynamic volume (V_h_) distribution using gel permeation chromatography (Figure 1A,B). This analysis enabled the assessment of differences between dissolved samples in terms of the molecular characterization. The esterification of starch with 1% OSA did not significantly affect the height of the peak of fraction with large hydrodynamic volumes (corresponding to amylopectin) (Figure 1A). However, a decrease was observed in the peak of the fraction with smaller hydrodynamic volumes (corresponding to amylose), accompanied by an increase in the content of fraction with the smallest hydrodynamic volumes.

This suggests that the lowest level of OSA used for starch modification led to a slight depolymerization of the amylose fraction. Similar observations were made by Simsek et al. [29]. The authors of this manuscript noticed that the molecular weights of amylose decreased significantly in OS potato starch with DS 0.024, while the molecular weights of amylopectin remained unchanged. In the remaining starches esterified using octenyl succinic anhydride, a significant decrease in the peak height of the starch fraction with the largest hydrodynamic volume was observed, along with a reduction in the area under the peak corresponding to the amylose fraction. These changes were accompanied by an increase in the fraction with hydrodynamic volumes ranging from 10^−19^ to 10^−22^ mL. The obtained results indicate that slight depolymerization occurred in both the amylose and amylopectin fractions in the samples of starch esterified with 3%, 5%, and 7% OSA. The slight depolymerization observed in the esterified starches was primarily attributed to structural reorganization and changes in granule compactness. In our previous study, where maize starch was used as the research material, some degradation of the fraction corresponding to amylopectin was also observed under the influence of a high concentration of OSA used for esterification [28]. Figure 1B presents the hydrodynamic volume distributions of native starch and the blank sample used in the study. The blank sample was prepared from native starch using the octenyl succinate starch preparation procedure but without the addition of the modifying reagent. Native potato starch exhibited a slightly higher proportion of the largest hydrodynamic volume fractions (around V_h_ = 10^−16^) compared to the blank sample. Treatment under the conditions using modification reduced the content of the fraction corresponding to amylopectin while increasing the fraction with smaller V_h_, corresponding to amylose. The OSA starch preparation was performed under alkaline conditions, and the reaction was terminated by acidification, followed by washing with water and alcohol. These steps subtly depolymerized the higher-volume fractions, affecting the hydrodynamic volume distributions of potato starch.

During production of mixtures with fatty acids, starches with granular structures were used. The complexing index (CI) was analyzed to determine the effect of OSA esterification on complex formation between starch and oleic acid during gelatinization (Table 2). The complexing index of starch with oleic acid increased linearly with the degree of starch substitution with octenyl succinate groups (determined correlation coefficient R^2^ = 0.987). The mixture of starch esterified with 7% OSA and oleic acid exhibited a fourfold higher complexing index compared to the counterpart prepared from non-esterified potato starch. Similar results published by Wang et al. [30] indicated that OSA modification substantially promoted complexation between starch and selected lipids (e.g., palmitic acid), with greater effects noted for octenyl succinate starches with higher degrees of substitution. Thus, it could be concluded that emulsifying activity of OSA-modified starch can promote its complex formation with fatty acids. Supplementary Figure S1A shows the relationship between the complexing index and the percentage of substitution with OS groups, revealing that higher substitution levels are associated with an increased complexing index.

The lipid content in the mixtures was approximately 3% and did not differ between starches with different octenyl succinate substitutions (Table 2). The lipid content in the examined mixtures was on the same level as determined in our previous study, where mixtures of acid-treated starch and oleic acid were produced [18].

#### 2.1.2. Water Binding Capacity and Solubility in Water

**Figure 2:**
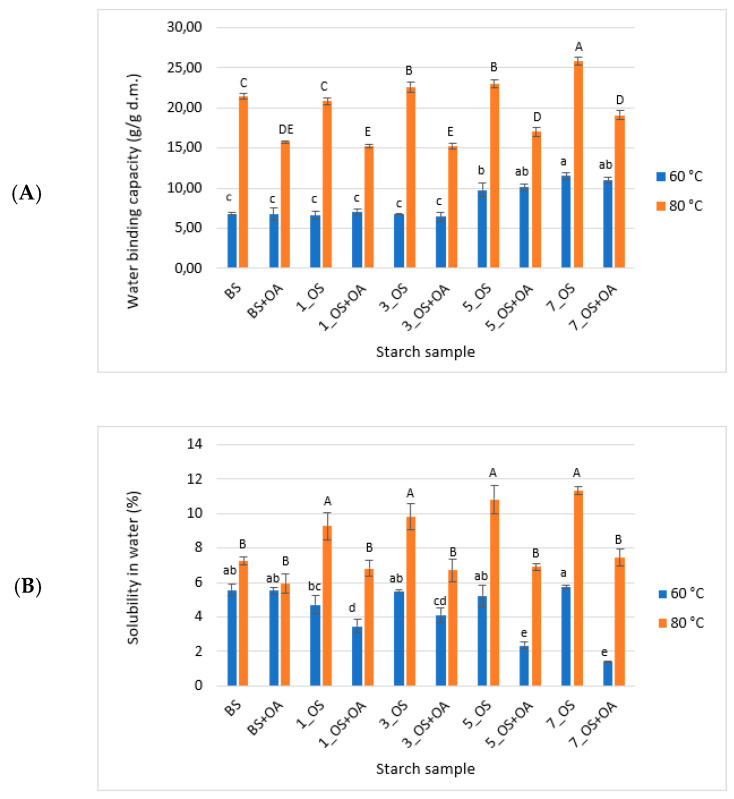
(**A**) Water binding capacity and (**B**) water solubility of tested octenyl succinate starches and their mixtures with oleic acid. BS: blank sample; 1_OS: octenyl succinate starch modified with 1% OSA; 3_OS: octenyl succinate starch modified with 3% OSA; 5_OS: octenyl succinate starch modified with 5% OSA; 7_OS: octenyl succinate starch modified with 7% OSA; BS+OA, 1_OS+OA, 3_OS+OA, 5_OS+OA, 7_OS+OA: corresponding starch samples mixed with oleic acid. Within series, columns with the same letter are not significantly different (α < 0.05) by Fisher’s least significant difference test.

Figure 2 presents the values of water binding capacity and water solubility of OS starch and its mixtures with oleic acid measured at 60 °C and 80 °C. Mixing OS starch with oleic acid did not significantly affect water binding capacity at 60 °C compared to the esterified counterpart, regardless of the degree of substitution. Among the studied starch derivatives, the highest water binding capacity at this temperature was exhibited by OS starches esterified at 5% and 7%, as well as their mixtures with oleic acid. At the measurement temperature of 80 °C, OS starches exhibited water binding capacity above 20 g/g d.m., with starch esterified with 7% OSA showing slightly higher values compared to other OS starches. The introduction of oleic acid to OS starches decreased water binding capacity at this temperature by approximately 30%. This effect may result from reduced water accessibility in the starch structure due to hydrophobic interactions between the hydrophobic OS groups in the starch and the long hydrocarbon chains of oleic acid, thereby decreasing the availability of water binding regions. In our previous study, a similar trend was observed: oleic acid significantly reduced the swelling power at 80 °C of starch derivatives and starch hydrolysates [18].

Esterification of starch with octenyl succinic anhydride did not affect the water solubility determined at 60 °C. However, the addition of oleic acid to OS starch resulted in a gradual decrease in values of this parameter. The highest inhibition of water solubility at 60 °C was observed in the starch esterified with 7% OSA mixed with oleic acid, which exhibited a fourfold lower value compared to its counterpart prepared without the acid addition. Slightly different trends were observed at 80 °C. In this case, esterification of starch with OSA resulted in a slight increase in water solubility compared to blank starch, with no significant effect of the degree of esterification. Mixing octenyl succinate with oleic acid resulted in a reduction in water solubility at 80 °C by more than 30% compared to its non-complexed counterpart. The most significant changes were observed in starches esterified with 5% and 7% OSA. These starches exhibited the highest complexation index after gelatinization. At a temperature of 80 °C, mixtures of starch and oleic acid underwent pasting, leading to the formation of amylose–lipid complexes, which significantly decreased starch solubility [31].

#### 2.1.3. Thermodynamic Characteristics of Gelatinization

The results of the thermodynamic analysis of starch gelatinization determined by differential scanning calorimetry (DSC) are presented in Table 3. The introduction of oleic acid into octenyl succinate starches exerted a subtle influence on the thermodynamic parameters of gelatinization. Decreases in the onset (T_o_) and peak (T_p_) were observed upon mixing oleic acid with starch esterified with 3% and 5% OSA. The most pronounced effect was found in the mixture prepared from starch with the highest degree of substitution, where the addition of oleic acid led to an approximately 6% increase in T_o_ compared with the control starch not combined with the lipid. This sample exhibited the highest complexation index. These complexes likely formed within the helical structure of amylose, stabilizing it, and require a higher temperature to initiate gelatinization, which explains the observed increase gelatinization temperatures [32]. Among the octenyl succinate starches investigated, the sample esterified at a 7% level exhibited slightly lower transition temperatures compared with the blank starch.

DSC measurements revealed a slight decrease in gelatinization enthalpy (ΔH) only for blank potato starch and for OS starch with the lowest degree of substitution as a result of oleic acid addition. During the thermodynamic analysis of gelatinization, ΔH reflects the energy required to disrupt hydrogen bonds and to break down the ordered crystalline structures of starch [33,34]. In blank or low substituted OS starch, the polymer structure is primarily stabilized by hydrogen bonding and other hydrophilic interactions. The addition of oleic acid may form local hydrophobic aggregates within the starch structure, reducing water accessibility to some of the hydrophilic areas and consequently decreasing the enthalpy of the gelatinization process. In contrast, OS starches with higher DS values did not exhibit significant changes in ΔH compared to their counterparts not mixed with oleic acid. The gelatinization enthalpy reflects the amount of energy required to disrupt the ordered structure within starch granules, mainly involving amylose and amylopectin. In the case of starch with a high degree of substitution, ΔH remained unchanged after the addition of oleic acid. This indicates that oleic acid likely forms complexes with the amylose, as reflected by the high complexation index (Table 2), but does not appear to significantly disrupt the internal structure of the granules.

#### 2.1.4. Pasting Characteristic

Table 4 presents the pasting characteristics of the studied mixtures of OS starch with oleic acid. Mixing OS starch with oleic acid significantly affected the parameters of the pasting profile.

The introduction of oleic acid into the OS starch structure reduced both the peak viscosity (PV) and hot paste viscosity at 95 °C (HPV) by approximately 60% compared to its non-mixed counterpart. This decrease is attributed to the ability of oleic acid to form inclusion complexes with amylose, thereby restricting its leaching during pasting, which in turn markedly reduces the viscosity of the resulting pastes [35]. The final viscosity (FV) values at 50 °C also decreased as a result of oleic acid addition. However, in this case, the trend was dependent on the degree of starch substitution with octenyl succinate groups. With an increasing substitution level, the inhibitory effect on final viscosity was also intensified. Generally, modification of starch through complexation with lipids limits the ability of amylose chains to associate and crystallize, which leads to a significant decrease in the final viscosity of starch pastes [36,37]. However, the results presented in the present study indicate that, during cooling, the reassociation of amylose chains into an ordered network was restricted by both oleic acid and the extent of substitution with OS groups. This phenomenon could be attributed to the fact that as the degree of substitution increases, the hydrophobic character of OS starch becomes more pronounced. This promotes stronger interactions between oleic acid and the starch matrix. During the peak viscosity measurements, all granules swell in a similar manner, and the influence of hydrophobic interactions is not evident at this stage. In the later stages of pasting, during the final viscosity measurements, local hydrophobic interactions become more significant, and the effect of the degree of substitution with OS is also observed.

Noteworthy relationships were observed in the esterified starch samples that were not subjected to mixing with oleic acid. The esterification of native starch with octenyl succinic acid resulted in a reduction of peak viscosity by 30% when a 1% concentration of the modifying agent was used, or by approximately 60% when higher doses of OSA were used in the reaction. It is worth noting that an increase in the OSA concentration from 3% to 7% did not affect the peak viscosity of the pastes. After reaching a certain level of DS, further increases did not lead to significant changes in this parameter. It has been previously reported that the maximum viscosity is mainly related to the swelling and solubility of starch granules [38]. In this study, a strong negative correlation (−0.91) was observed between the values of solubility in water at 80 °C and the peak viscosity of OS starch pastes, which confirms this statement. This finding indicates that higher OS starch solubility results in a lower number of swollen granules, which consequently lead to a lower maximum paste viscosity. Supplementary Figure S1B presents the relationship between maximum viscosity of octenyl succinate starch pastes and their water solubility at 80 °C, showing a negative correlation between these parameters.

The pastes prepared from mixtures of oleic acid with OS starch exhibited low breakdown values (BD), oscillating around 20 m·Pas. This indicates that the difference between peak viscosity and hot paste viscosity in these samples was small, which reflects the high stability of the paste during heating and shearing. However, the overall viscosity values were low, and the pastes formed weak gels. The addition of oleic acid to OS starch influenced the setback parameter (SB) differently depending on the degree of substitution. SB represents the tendency of amylose chains to retrograde or re-associate during cooling [39]. In the case of pastes prepared from starches with higher DS values, the reduction in SB can be attributed to more extensive starch–lipid associations, which stabilize the granule structure, restrict amylose leaching, and consequently reduce the extent of retrogradation.

RVA measurements revealed that the pasting temperature (P_t_) increased by about 20% in starch–oleic acid mixtures compared to the corresponding starches without addition of oleic acid. The greatest increase was observed in the mixture prepared with starch esterified with 5% of OSA. This increase can be attributed to starch–lipid interactions that hinder granule swelling and delay amylose release; therefore, a higher temperature was required to initiate pasting.

#### 2.1.5. Surface and Interfacial Tension

**Figure 3:**
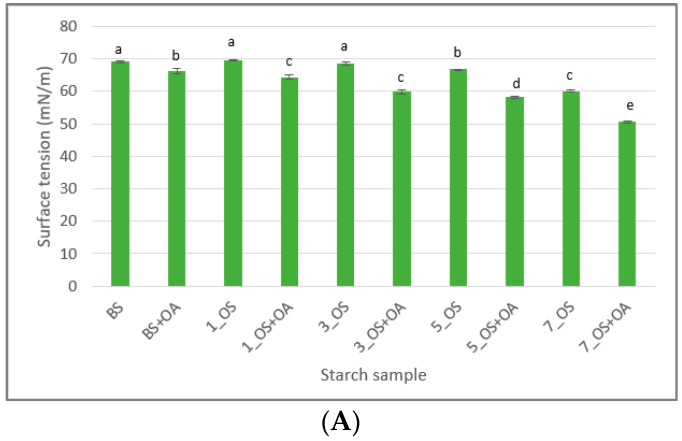

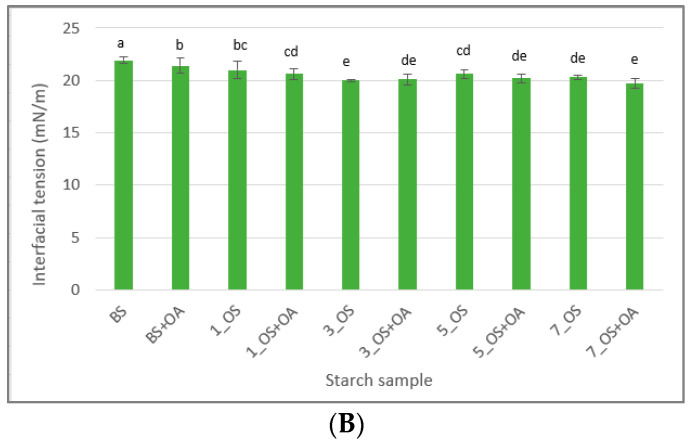
(**A**) Surface and (**B**) interfacial tensions of tested octenyl succinate starches and their mixtures with oleic acid. BS: blank sample; 1_OS: octenyl succinate starch modified with 1% OSA; 3_OS: octenyl succinate starch modified with 3% OSA; 5_OS: octenyl succinate starch modified with 5% OSA; 7_OS: octenyl succinate starch modified with 7% OSA; BS+OA, 1_OS+OA, 3_OS+OA, 5_OS+OA, 7_OS+OA: corresponding starch samples mixed with oleic acid. Columns with the same letter are not significantly different (α < 0.05) according to Fisher’s least significant difference test.

Surface and interfacial tensions of tested octenyl succinate starches and their mixtures with oleic acid are presented in Figure 3. Esterification of potato starch with octenyl succinic acid at levels of 5% and 7% resulted in a reduction of the surface tension of the pastes obtained from these starches, with a more pronounced effect observed in the sample with a higher degree of substitution. Mixing OS starch with oleic acid led to a reduction in the surface tension of the pastes compared to the counterpart without the acid addition. The influence of the degree of substitution with OS groups on the reduction of the surface tension of pastes containing added oleic acid was observed. In the sample esterified with 1% OSA, the addition of oleic acid reduced the surface tension by 7%, while in the sample with the highest degree of substitution, the reduction reached 16%.

In general, esterification introduced octenyl succinate groups into the starch structure, thereby imparting surface-active properties to the polymer [1]. A higher degree of substitution corresponds to a greater number of such groups, resulting in a stronger reduction of surface tension (within the range of the studied degrees of substitution). In addition, the introduced hydrophobic chain of oleic acid may interact with the OS groups in the starch, primarily through hydrophobic interactions involving van der Waals forces or associative effects. From this perspective, the addition of oleic acid enhances the surface tension lowering effect, leading to the formation of a more stable and efficient surfactant system at the phase interface.

In contrast, the addition of oleic acid to OS starch did not affect the interfacial tension values, whereas a slight decrease was observed upon addition of oleic acid to blank starch. The introduction of oleic acid to OS starch did not alter the interfacial tension values, probably because the interfacial region was already covered with amphiphilic octenyl succinate groups covalently bound to the starch. These groups efficiently adsorb at the oil–water interface and provide stable coverage. Unlike OS starch, blank starch does not contain surface-active substituents. Under these conditions, oleate ions can adsorb at the interface, which accounts for the subtle decrease in interfacial tension. These findings are consistent with previous studies on the interfacial behavior of OS starch and different oils. Zhao et al. [40] showed that hydrophobic OS starch readily diffuses to and adsorbs onto oil surfaces, such as palm oil, reducing interfacial tension and stabilizing the interface. This supports the idea that the amphiphilic octenyl succinate groups on OSA starch dominate interfacial adsorption, whereas blank starch relies on the adsorption of added surfactants such as oleate.

### 2.2. Evaluation of Films Properties

**Figure 4:**
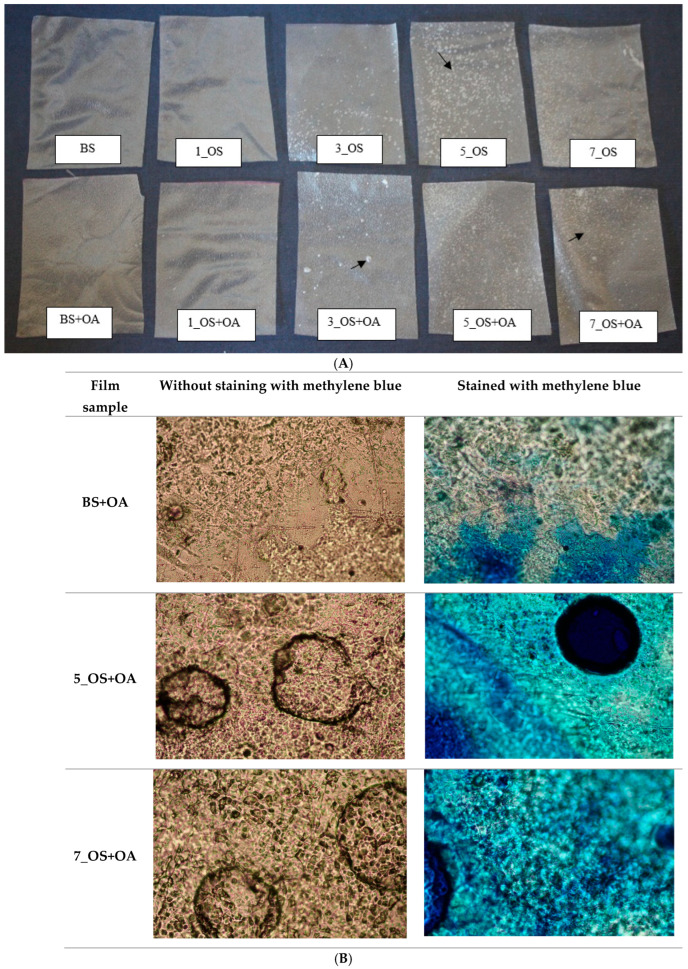
(**A**) Appearance of tested edible films prepared from octenyl succinate starches and their mixtures with oleic acid. (**B**) Optical micrographs of tested edible films prepared from octenyl succinate starches and their mixtures with oleic acid with or without staining with methylene blue. BS: blank sample; 1_OS: octenyl succinate starch modified with 1% OSA; 3_OS: octenyl succinate starch modified with 3% OSA; 5_OS: octenyl succinate starch modified with 5% OSA; 7_OS: octenyl succinate starch modified with 7% OSA; BS+OA, 1_OS+OA, 3_OS+OA, 5_OS+OA, 7_OS+OA: corresponding starch samples mixed with oleic acid.

Figure 4A presents the appearances of the tested edible films. Additionally, selected films were analyzed using an optical microscope (Figure 4B). The tested films, assessed visually, were transparent, colorless, and odorless. The potato starch film was characterized by the most homogeneous surface. Unfortunately, in the case of the films prepared from OS starch, the polymer network contained microbubbles (examples indicated by a black arrow). They were likely formed as a result of foaming of the starch paste during mixing. This phenomenon was facilitated by the reduced surface tension of the OS starch, which promoted bubble formation and stabilization within the film structure. The presence of microcracks and bubbles in the structure of films prepared from OSA starch was also proven in our previous study on OS starch-based films [24]. Additionally, films prepared from starches containing oleic acid were stained with methylene blue to differentiate areas where the oil fraction was irregularly distributed within the starch–glycerol matrix. In Figure 4B, these areas appear as bright regions surrounding the blue-stained hydrocolloid phase. Areas of coalesced lipid were observed in the film prepared from a mixture of blank starch with oleic acid. In this sample, no stabilizing agent was present to maintain oil dispersion. As a result, after mixing the film-forming solution, the liquid oleic acid droplets coalesced and were not uniformly distributed across the film surface. In contrast, films prepared from starch octenyl succinate derivatives with the addition of oleic acid revealed a uniform blue coloration without bright areas after staining with methylene blue. This indicates that oleic acid was evenly dispersed throughout the film surface. This effect was attributed to the amphiphilic groups of the octenyl succinate, which stabilized the starch–lipid system.

#### 2.2.1. Water Vapor Permeability of the Films (WVP)

The obtained starch films were subjected to analysis of water-related properties (Table 5). Films prepared from blank starch exhibited the lowest water vapor permeability, while the addition of oleic acid slightly increased values of this parameter. The introduction of hydrophobic OS groups increased permeability compared to BS. The addition of oleic acid to starch esterified with OSA at amounts above 1% led to a decrease in water vapor permeability of obtained films (in the case of the film based on starch modified with 5% OSA, the change was not statistically significant).

The addition of oleic acid to blank starch led to increased water vapor permeability. This is likely due to oleic acid droplet coalescence, which caused a less uniform and more heterogeneous film structure, facilitating water transport through the material. Surprisingly, films prepared from octenyl succinate starch exhibited higher water permeability compared to films made from blank potato starch. As mention above (Figure 4A,B), the polymer network of OS starch films included microbubbles. The presence of these small bubbles leads to the formation of micropores in the film structure, significantly facilitating water transport through the material. These results suggest that, in the case of octenyl succinate starch films, the chemical hydrophobicity effect (arising from the presence of OS groups in the starch) may be outweighed by the structural defects in the matrix caused by the presence of air bubbles. A slight increase in water permeability of films prepared from OS potato starch was also observed in our previous study [24]. This study confirmed the impact of both the quantitative ratio of hydrophilic to hydrophobic constituents in the film and microstructure of the OS starch films on water vapor permeability. At higher degrees of OS substitution, the presence of oleic acid enhances the hydrophobicity of the film matrix, restricting water migration as indicated by a reduction in WVP. The emulsifying function of OS starch results from the presence of hydrophobic octenyl succinate groups incorporated into the starch chain, which stabilize the oil–water interface and prevent droplet coalescence [41]. These observations were also confirmed in our previous studies [42]. Due to the presence of OS groups, the oleic acid was uniformly dispersed within the film matrix, preventing droplet coalescence. The well-dispersed oleic acid promoted the formation of more uniform and hydrophobic regions within the film, resulting in reduced water vapor permeability.

#### 2.2.2. Water Binding Capacity and Water Solubility

Notably, the incorporation of oleic acid into OS starch resulted in a considerable reduction in the water binding capacity of the films (Table 5). This effect was most evident in films prepared from mixtures of oleic acid and starch esterified with 5% or 7% OSA. Film prepared from OS starch with the highest degree of substitution exhibited extremely high water binding capacity, absorbing so much water that measuring its mass could not be performed. Following modification with oleic acid, these films showed markedly low water binding. Similarly, in films based on starch esterified with 5% OSA, the addition of oleic acid reduced water binding capacity by nearly 60%, demonstrating a strong inhibitory effect of oleic acid on water uptake. The water binding capacity of the films prepared from octenyl succinate starches without the addition of oleic acid was strongly influenced by the degree of substitution. Films prepared from starches esterified with 5% and 7% OSA showed increased water binding ability compared to those from blank starch. It was probably due to the fact that hydrophobic interactions between OS groups locally grouped the chains of starch, creating more compact regions within the film matrix. Consequently, it modified the organization of the remaining hydrophilic chains, creating spaces into which water could penetrate. This demonstrates that water binding depends not only on chemical composition but also on the structural organization of the film matrix. Similarly, Naseri et al. [43] showed that modification of sago starch with OSA increased water adsorption as well as water vapor permeability of starch films. According to these authors, the introduction of OS groups into starch creates larger spaces within the polymer matrix of the film, providing adsorption space and allowing water to pass.

The water solubility of the films was also evaluated (Table 5). In most samples, esterification of potato starch with octenyl succinic anhydride had no significant effect on film solubility, with the exception of starch modified at the 7% OSA level, where a slight increase was observed. This suggests that the introduction of OS groups into the starch structure did not restrict the solubility of the films. In contrast, the introduction of oleic acid reduced the solubility of the films by approximately 20%. The strongest inhibitory effect of oleic acid was found in films prepared from starch esterified with 5% OSA.

In general, the hydrophilic nature of starch films is attributed to the network formed by amylose and amylopectin molecules stabilized by hydrogen bonding [44]. Although OS starch is amphiphilic, it does not significantly reduce film solubility. This demonstrates that hydrophilic network interactions are dominant in the film network. In contrast, the introduction of oleic acid to OS starch restricts water accessibility, decreasing solubility of the films. In this context, the amphiphilic character of OS starch facilitates the stabilization of hydrophobic interactions with oleic acid, enhancing the observed effect on water resistance. The enhanced water resistance of films prepared from starches combined with oleic acid was demonstrated by a significant reduction in their water binding capacity, compared with both the unmixed OS counterparts and the blank starch films. The most pronounced effects were observed in the 5% and 7% OS starch films, where the addition of oleic acid lowered water binding capacity by up to 60%. Even in the mildly modified 1_OS+OA sample, this parameter was half that of the blank film. Although changes in water solubility were less pronounced, a similar decreasing trend was observed. The incorporation of oleic acid reduced solubility values by approximately 20% relative to OS-modified films and by 10–24% relative to blank films. Taken together, these results indicate that the combined treatment with OSA and oleic acid can meaningfully improve the water resistance of the films, even if the water vapor permeability was less strongly affected. Those results are highly noteworthy as they offer a valuable approach to enhancing the functional properties of starch-based materials.

## 3. Materials

Potato starch (Superior Standard LU-1431-1) was obtained from WPPZ SA (Luboń, Poland). High-purity octenyl succinic acid anhydride was supplied from Sigma-Aldrich Chemical Co. (St. Louis, MO, USA) and pure oleic acid (WE-204 007-1) from Chempur (Piekary Śląskie, Poland). The other chemicals used in the study were of analytical purity.

### 3.1. Preparation of Octenyl Succinate Starches

OS starch samples were prepared according to Hui et al. [27]. Starch (300 g, d.m.) was dispersed in distilled water under continuous stirring to obtain a 35% (*w*/*w*) starch suspension. The pH of the suspension was adjusted to 8.0 using a 3% (*w/v*) NaOH solution. The OSA solution, diluted fivefold with absolute ethanol, was gradually added to the starch suspension over 1 h. For esterification, different amounts of OSA, 1%, 3%, 5%, 7% (*v/v*, based on d.m. of starch), were used. The reaction was then processed for 3.5 h under constant stirring. Afterwards, the pH was lowered to 6.5 with 3% (*v/v*) HCl, and the mixture was centrifuged and washed twice—first with distilled water and then with 70% aqueous ethanol. The resulting solid was dried in an oven at 45 °C for 24 h and sieved through a 125 μm mesh. Obtained OS starches were marked as 1_OS, 3_OS, 5_OS, and 7_OS, respectively. To obtain a blank sample (BS), the same process was carried out without any addition of OSA to the starch solution.

### 3.2. Production of OS Starch–Oleic Acid Mixtures

OS starch with oleic acid mixtures were prepared according to Wang et al. [17]’s procedure previously reported in our earlier paper [18]. A total of 1.5 mmol of oleic acid was dissolved in 30 mL of ethanol under magnetic stirring, then mixed with 10 g of dry starch (BS or OS starches). The obtained starch–fatty acid mixtures marked as BS+OA, 1_OS+OA, 3_OS+OA, 5_OS+OA, and 7_OS+OA were stored at 4 °C until further use. The complexing index of oleic acid in OS starches was determined according to Wang et al. [17].

### 3.3. Formation of Edible Films Based on OS Starches and Their Mixtures with Oleic Acid

To obtain edible films, the method developed by Osés et al. [45] was used. A total of 350 g of aqueous suspension of starch derivative at a concentration of 2 % (*w/w*, based on d.m. of starch) was preheated at 95 °C for 30 min under a stirring speed of 300 rpm. Then, glycerol with relative weights of 30% (with regard to the d.m. of starch) was added as a plasticizer, and the whole mixture was mixed for an additional 10 min. Film-forming solution was poured into a rectangular tray (33 cm × 25 cm) and was dried at 60 °C and relative humidity (RH) = 60% in a dryer Venticell Standard (BMT, Brno, Czech Republic). Finally, film was removed from the trays and was stored in a desiccator at a 20 ± 2 °C and 50 ± 2% RH before further analysis.

## 4. Methods

### 4.1. Effectiveness of Modification Procedures

#### 4.1.1. Degree of Substitution

The degree of substitution of OS starches with octenyl succinate groups was determined according to the method of Hui et al. [27]. For this, 5 g of OS starch (d.m.) was stirred in 25 mL of a 2.5 M HCl isopropyl alcohol solution for 30 min. Then, 100 mL of 90% (*v/v*) aqueous isopropyl alcohol was added, and the mixture was stirred for an additional 10 min. The suspension was filtered through a glass filter, and the residue was washed repeatedly with 90% aqueous isopropyl alcohol until no chloride ions were detectable. The washed starch was re-dispersed in 300 mL of distilled water and heated in a boiling water bath for 20 min. The resulting solution was titrated with 0.1 M NaOH using phenolphthalein as an indicator. Native starch was titrated in parallel as a blank. The DS value and the percentage of substitution (%OS) were then calculated using the following formulas (Equations (1) and (2)).

(1) $$DS=\frac{0.162\cdot (A\cdot M)/W}{1-\left[0.210\cdot (A\cdot M)/W\right]}$$

(2) $$\%OS=\frac{210\cdot DS}{162+\left(210\cdot DS\right)}\cdot 100\left[\%\right]$$

where *A* is the titration volume of NaOH solution (mL), *M* is the molarity of NaOH solution, and *W* is the dry weight (g) of starch.

#### 4.1.2. Determination of Complexing Index

The CI of starch was measured by the method of Wang et al. [46] with the following modifications. Starch or starch–fatty acid mixtures (0.4 g) were weighted and mixed with 20 mL of distilled water. Then, the starch suspension was heated in a boiling water bath for 10 min with shaking. After cooling to room temperature, 500 µL of the paste was taken and mixed with 15 mL of distilled water and 500 µL of iodine solution (0.1% KI and 0.026% I_2_ in distilled water). The absorbance at λ = 621 nm was measured. The complexing index was calculated as follows (Equation (3)):

(3) $$CI=\frac{{Abs}_{strch}-{Abs}_{starch-lipid}}{{Abs}_{starch}}[\%]$$

where *Abs_starch_* was the absorbance of the starch without added oleic acid solution, and *Abs_starch–lipid_* was the absorbance of the starch with oleic acid mixture solution.

#### 4.1.3. Determination of Total Lipid Content

The total lipid content of blank and OS starches, as well as their mixtures with oleic acid, was measured using Soxhlet extraction according to the standard analytical method PN-EN-ISO 3947:2001 [47]

#### 4.1.4. Determination of Equivalent Hydrodynamic Volume Distribution

The determination of the equivalent hydrodynamic volume distribution of blank and OS starches was conducted according to the method described in our previous paper by Królikowska et al. [2]. A gel permeation chromatograph (GPC) equipped with a Smartline 100 HPLC pump (Knauer, Berlin, Germany), two columns OHpak SB-806 and OHpak SB-805 (Shodex, Tokyo, Japan), and a refractive index detector Smartline 2300 (Knauer, Berlin, Germany) was used. The eluent phase was 0.1 mol/L water solution of sodium nitrate containing 0.2 g/L sodium azide. The temperature of columns was maintained at 25 °C; the flow rate of the mobile phase was set at 0.5 mL/ min. Pullulans (Standard P-82, Shodex, Tokyo, Japan) were used as standards. The distribution of equivalent hydrodynamic volume, which is closely related to the size of dissolved polymer chains and gives information about the composition and structure of polymer molecules in solution, was calculated according to Vilaplana & Gilbert [48] and Coseri et al. [49].

### 4.2. Characterization of Octenyl Succinate Starches and Their Mixtures with Oleic Acid

#### 4.2.1. Water Binding Capacity (WBC) and Solubility in Water (SW)

The water binding capacity and solubility in water were determined according to the method of Li et al. [50]. A starch sample (0.15 g, d.w.) was weighed (*W*_0_) into a pre-weighed centrifuge tube with a screw cap, and 10 mL of deionized water was added. The starch suspension was heated with constant stirring over 1 h at temperatures of 60 °C or 80 °C. Afterwards, the samples were rapidly cooled down in a water bath and then centrifuged at 3000× *g* for 30 min. To determine solubility in water, the supernatant was carefully transferred into a weighing vessel, evaporated, and dried at 100 °C until the constant weight was obtained (*W_S_*). To determine water binding capacity, the residue remaining in the centrifuge tube was also weighed (*W*_1_). The water binding capacity and solubility in water were calculated as follows (Equations (4) and (5)):

(4) $$SW=\frac{W_{1}}{W_{0}}\cdot 100\;[\%]$$

(5) $$WBC=\frac{W_{S}}{W_{0}\cdot \left(100-SW\right)}[\frac{g}{g}d.w.]$$

#### 4.2.2. Thermodynamic Characteristics of Gelatinization

The gelatinization parameters of starches and their mixtures with oleic acid were measured using a differential scanning calorimeter DSC 40000 (Perkin Elmer, Shelton, CT, USA). A starch derivative–water mixture (1:3) was placed into an aluminum calorimetric pan and left to stand for 24 h. The sample was heated in a temperature range of 30 to 100 °C at a rate of 10 °C/min. An empty calorimetric pan was used as a reference. The onset, peak, and end (T_e_) temperatures, as well as the enthalpy of gelatinization, were calculated.

#### 4.2.3. Pasting Characteristic

Pasting properties were studied on 5% *w/w* starch suspension using Rapid Visco Analyzer (RVA) (Perten Instruments, Warriewood, Australia). The sample was held at 50 °C for 1 min, heated to 95 °C for 5.5 min, and held at 95 °C for 5 min. Then, it was cooled to 50 °C for 5 min and held at that temperature for another 5 min. The results were interpreted with respect to pasting temperature, peak viscosity, hot paste viscosity at 95 °C, final viscosity at 50 °C, breakdown value (BD = PV−HPV), and setback value (SB = FV−HPV).

#### 4.2.4. Surface and Interfacial Tensions of Starch and Mixture Pastes

The water suspensions of blank and OS starches and their mixtures with oleic acid at a concentration of 1% *w/w* were pasted at 95 °C under constant stirring and then cooled to a temperature of 23 °C ± 1 °C. The densities of the obtained pastes, water, and oil were determined using a Densito 30 P oscillating densimeter (Mettler-Toledo GmbH, Greifensee, Switzerland). The surface and interfacial tensions of the tested pastes were measured as described by Lima et al. [51] with modification. The evaluations were performed using the pendant drop method with an OCA goniometer (Dataphysics Instruments GmbH, Filderstadt, Germany) equipped with an electronic injection system and a CCD video camera with a resolution of 768 × 576 pixel. During surface tension measurements, a drop of paste (8 µL) was formed using a syringe (Hamilton 5 mL) and suspended at the tip of a stainless-steel needle with an internal diameter of 0.525 mm; here, it was digitally captured by camera. For the measurement of the internal tension in the water-in-oil system, a paste drop of volume 12 µL was injected into an optical glass cuvette filled with oil. The contour of the drop was analyzed using image analysis software SCA 20 (Dataphysics Instruments GmbH, Filderstadt, Germany) to infer the surface tension. Tests were performed at 22–24 °C.

### 4.3. Evaluation of the Functional Properties of the Films Prepared from Octenyl Succinate Starches or Their Mixtures with Oleic Acids

#### 4.3.1. Microscopy Observation of Emulsions

Photo images and micrographs of films were taken using a digital camera (Digital Ixus 100 IS, Canon, Tokyo, Japan). Optical micrographs of the films were captured under an Eclipse E200 optical microscope (Nikon, Tokyo, Japan) at room temperature. A piece of film was placed onto the microscope slide and carefully covered. Selected films prepared from OS starch mixed with fatty acids were photographed after staining with methylene blue. Images were observed at 10x magnification.

#### 4.3.2. Determination of Water Binding Capacity and Water Solubility

Water binding capacity and solubility in water of the tested films were determined and calculated according to modified method described by Ryu et al. [52] and Šuput et al. [53]. Film samples (20 × 20 mm) were weighed (*M*_1_), dried at 100 °C for 24 h, and reweighed to obtain the initial dry mass (*DM*_0_). The pieces were then immersed in 50 mL of distilled water in 100 mL conical flasks, sealed, and kept in a shaking water bath at 25 °C for 24 h. Afterwards, the content was filtered through qualitative filter paper and weighed in the swollen state (*M*_2_). The samples were subsequently dried again at 100 °C for 24 h to determine the final dry mass (*DM*_24_). Water binding capacity and water solubility were calculated as follows (Equations (6) and (7)):

(6) $$Solubility=100\times \frac{{DM}_{0}-{DM}_{24}}{{DM}_{0}}\left[\%\right]$$

(7) $$Water\;binding\;capacity=100\times \frac{M_{2}-{DM}_{24}}{{DM}_{24}}\left[\%\right]$$

#### 4.3.3. Water Vapor Permeability

Water vapor permeability was determined following ASTM E96 [54] with modifications. Circular film specimens (3 cm diameter) were cut, and their average thickness was measured. The films were sealed over cups filled with distilled water, leaving a 2 cm gap between the water surface and the film. The cups were placed in a desiccator with magnesium nitrate-Mg(NO_3_)_2_ to maintain RH = 53 ± 2% and stored in a dryer at 32 °C for 9 days. After a 48 h equilibration period, cup weights were measured twice daily. The slope of the line of mass change over time (R^2^ > 0.99) was used to calculate WVP (Equation (8)).

(8) $$WVP=\frac{a}{FA}\times \frac{L}{{PA}_{1}{-PA}_{2}}\left[g/s\cdot m\cdot kPa\right]$$

where *a*—slope of the line (g/s); *FA*—film area (m^2^); *PA*_1_—vapor partial pressure at film outer surface in the cabinet (kPa); *PA*_2_—vapor partial pressure at film inner surface in cup (kPa); and *L*—the average film thickness (m).

### 4.4. Statistical Analysis

All analyses were replicated at least three times, and all data are reported as mean ± SD. Data were statistically handled by one-way analysis of variance (ANOVA) using Statistica 12 software. Fisher’s test was applied for the calculation of the significant differences among the values of characteristic parameters at probability level α≤ 0.05. Pearson correlation coefficients were calculated to assess the relationships between selected variables. The relationships between selected parameters showing significant correlations are presented on the radar chart. The data were normalized.

## 5. Conclusions

The starch–oleic acid complexation index increased linearly with the rise in the degree of substitution, with the mixture prepared from 7% OSA-esterified starch exhibiting a fourfold higher complexation index compared to blank starch. The addition of oleic acid to octenyl succinate starches reduced their water binding capacity and solubility, especially at higher temperatures of determination (80 °C), which is associated with the formation of amylose–lipid complexes. Oleic acid also slightly altered the gelatinization of OS starch, decreasing T_o_ and Tₚ in low DS samples, while in high DS starch, it promoted the formation of amylose–lipid complexes without significantly changing the gelatinization enthalpy. The results also indicate that higher water binding capacity was associated with lower gelatinization temperatures and transition enthalpies. The addition of oleic acid to OS starch markedly reduced peak, hot paste, and final viscosities due to the formation of amylose–lipid complexes. As the degree of substitution increased, starch more effectively interacted with oleic acid, limiting amylose leaching and granule swelling. Pasting temperatures of starch–oleic acid mixtures increased by 20%, reflecting that additional energy was required to initiate pasting in the presence of lipids. The addition of oleic acid enhanced the surface tension lowering effect by interacting with the OS groups, forming a more stable surfactant system. OSA-esterified starch mixed with oleic acid reduced water vapor permeability of the films, except for the films based on starch esterified at 1% and 5%, where the addition of oleic acid did not result in a statistically significant reduction of WVP. It was due to enhanced hydrophobicity and uniform dispersion of the oleic acid within the starch matrix. In contrast, the presence of oleic acid increased water permeability in films prepared from blank starch. In these samples, coalescence of lipids occurred, resulting in a less uniform structure of the film. The incorporation of oleic acid into OS starch films markedly reduced their water binding capacity, particularly in films prepared from starch esterified with 5% or 7% OSA. Oleic acid also decreased the films’ solubility, with the strongest inhibitory effect observed in films from starch esterified with 5% OSA, indicating restricted water accessibility. These results demonstrate that the amphiphilic nature of OS starch stabilizes interactions with oleic acid, thereby significantly altering the functional properties of starch-based films. While WVP showed only minor differences among the samples, the addition of oleic acid markedly reduced solubility and water binding capacity, indicating an improvement in the water resistance of the films. The results indicate that octenyl succinate-modified starch, in combination with oleic acid, exhibits significantly different structural, thermal, and functional properties compared to blank starch. 

This study addressed key questions regarding the interactions of OS starch with lipids such as oleic acid. The obtained results systematically explore OS starch–oleic acid mixtures, characterizing their structural properties, the influence of the degree of substitution on complex formation, pasting behavior, and water binding capacity, as well as the development of starch–lipid films with improved water resistance. These findings provide a significant contribution to the understanding of OS starch–lipid interactions and their potential applications in food and material science. Mixtures of OS starch with oleic acid may have potential applications in various fields. Firstly, they can be used as stabilizers for emulsions and water-rich products. In foodstuffs such as sauces and dressings, surface-active properties of these mixtures can be desirable. Secondly, the presence of oleic acid alters the textural and rheological properties of OS starch, which can be utilized to design food products with specific consistency, such as creams and puddings. Additionally, OSA starch with oleic acid mixtures represent a promising component of coating matrices for bioactive lipid compounds including phospholipids and tocopherols. The films produced from studied mixtures, due to their enhanced resistance to water permeability, represent a promising material for packaging fruits, vegetables, cheeses, or snacks, protecting them from drying out and extending their shelf life.

## Figures and Tables

**Table 1:** Degree of substitution of tested octenyl succinate starches.

Starch Derivative	Degree of Substitution (-)	Substitution (%)
1_OS	0.0043 ± 0.0005 ^d^	0.60 ± 0.06 ^d^
3_OS	0.0092 ± 0.0007 ^c^	1.21 ± 0.08 ^c^
5_OS	0.0156 ± 0.0003 ^b^	2.02 ± 0.03 ^b^
7_OS	0.0217 ± 0.0003 ^a^	2.71 ± 0.05 ^a^

1_OS: octenyl succinate starch modified with 1% OSA; 3_OS: octenyl succinate starch modified with 3% OSA; 5_OS: octenyl succinate starch modified with 5% OSA; 7_OS: octenyl succinate starch modified with 7% OSA. Means within a column with the same letter are not significantly different (α < 0.05) by Fisher’s least significant difference test.

**Table 2:** Values of complexing index and lipid content in tested mixtures of octenyl succinate starches with oleic acid.

Starch Derivative	Complexing Index (%)	Lipid Content (%)
BS+OA	4.53 ± 0.40 ^e^	2.80 ± 0.10 ^a^
1_OS+OA	5.69 ± 0.49 ^d^	2.87 ± 0.12 ^a^
3_OS+OA	8.16 ± 0.14 ^c^	2.74 ± 0.09 ^a^
5_OS+OA	15.57 ± 0.27 ^b^	2.98 ± 0.01 ^a^
7_OS+OA	18.89 ± 0.40 ^a^	2.83 ± 0.16 ^a^

BS+OA: blank sample mixed with oleic acid; 1_OS+OA: octenyl succinate starch modified with 1% OSA mixed with oleic acid; 3_OS+OA– octenyl succinate starch modified with 3% OSA mixed with oleic acid; 5_OS+OA: octenyl succinate starch modified with 5% OSA mixed with oleic acid; 7_OS+OA: octenyl succinate starch modified with 7% OSA mixed with oleic acid. Means within a column with the same letter are not significantly different (α < 0.05) by Fisher’s least significant difference test.

**Table 3:** Thermodynamic characteristics of gelatinization of tested octenyl succinate starches and their mixtures with oleic acid.

Starch Derivative	T_o_ (°C)	T_p_ (°C)	T_e_ (°C)	ΔH (J/g)
BS	61.31 ± 0.03 ^cd^	65.79 ± 0.15 ^d^	72.67 ± 0.20 ^ab^	13.90 ± 0.36 ^a^
BS+OA	61.48 ± 0.06 ^bc^	66.08 ± 0.08 ^bc^	72.44 ± 0.12 ^bc^	12.35 ± 0.56 ^bcd^
1_OS	61.23 ± 0.28 ^cd^	65.80 ± 0.18 ^cd^	72.48 ± 0.31 ^ab^	13.36 ± 0.48 ^a^
1_OS+OA	61.03 ± 0.08 ^de^	65.81 ± 0.01 ^cd^	72.10 ± 0.19 ^cd^	12.61 ± 0.86 ^bcd^
3_OS	62.01 ± 0.06 ^a^	66.52 ± 0.08 ^a^	72.51 ± 0.06 ^ab^	12.79 ± 0.11 ^bc^
3_OS+OA	61.68 ± 0.14 ^b^	66.25 ± 0.17 ^ab^	72.42 ± 0.21 ^bc^	12.11 ± 0.91 ^cd^
5_OS	61.25 ± 0.24 ^cd^	66.39 ± 0.24 ^a^	72.82 ± 0.27 ^a^	12.13 ± 0.35 ^cd^
5_OS+OA	60.68 ± 0.11 ^f^	65.85 ± 0.11 ^cd^	71.88 ± 0.12 ^de^	11.79 ± 0.42 ^d^
7_OS	57.18 ± 0.24 ^g^	64.24 ± 0.25 ^e^	71.05 ± 0.22 ^f^	12.05 ± 0.26 ^cd^
7_OS+OA	60.80 ± 0.34 ^ef^	65.67 ± 0.24 ^d^	71.73 ± 0.21 ^e^	12.30 ± 0.11 ^cd^

T_o_: onset; T_p_: peak; T_e_: end temperature; ΔH: enthalpy of gelatinization; BS: blank sample; 1_OS: octenyl succinate starch modified with 1% OSA; 3_OS: octenyl succinate starch modified with 3% OSA; 5_OS: octenyl succinate starch modified with 5% OSA; 7_OS: octenyl succinate starch modified with 7% OSA; BS+OA, 1_OS+OA, 3_OS+OA, 5_OS+OA, 7_OS+OA: corresponding starch samples mixed with oleic acid. Means within a column with the same letter are not significantly different (α < 0.05) by Fisher’s least significant difference test.

**Table 4:** Pasting characteristics of tested octenyl succinate starches and their mixtures with oleic acid.

Starch Derivative	PV	HPV	BD	FV	SB	P_t_
	(mPa·s)	(mPa·s)	(mPa·s)	(mPa·s)	(mPa·s)	(°C)
BS	3969 ± 194 ^a^	1385 ± 28 ^c^	2584 ± 175	1693 ± 16 ^d^	308 ± 17	65.2 ± 0.1 ^c^
BS+OA	983 ± 4 ^d^	893 ± 22 ^f^	90 ± 19	1421 ± 15 ^e^	528 ± 13	67.2 ± 0.6 ^c^
1_OS	2750 ± 70 ^b^	1600 ± 56 ^a^	1150 ± 81	2133 ± 16 ^b^	533 ± 47	64.4 ± 1.2 ^c^
1_OS+OA	996 ± 7 ^d^	980 ± 17 ^e^	16 ± 11	1715 ± 49 ^d^	735 ± 32	73.0 ± 3.5 ^b^
3_OS	1703 ± 33 ^c^	1305 ± 47 ^d^	398 ± 80	2150 ± 19 ^b^	845 ± 29	65.7 ± 0.1 ^c^
3_OS+OA	637 ± 17 ^e^	618 ± 18 ^g^	18 ± 3	946 ± 26 ^f^	327 ± 16	74.6 ± 4.6 ^b^
5_OS	1696 ± 20 ^c^	1633 ± 16 ^a^	63 ± 3	2204 ± 26 ^a^	571 ± 11	65.7 ± 0.1 ^c^
5_OS+OA	568 ± 9 ^e^	541 ± 10 ^h^	27 ± 1	861 ± 13 ^g^	320 ± 4	81.2 ± 2.1 ^a^
7_OS	1619 ± 29 ^c^	1488 ± 23 ^b^	131 ± 8	1926 ± 30 ^c^	438 ± 8	65.2 ± 0.5 ^c^
7_OS+OA	644 ± 14 ^e^	618 ± 15 ^g^	25 ± 1	968 ± 14 ^f^	349 ± 1	75.9 ± 6.5 ^b^

PV: peak viscosity; HPV: hot paste viscosity (at 95 °C); BD = PV−HPV: breakdown; FV: final viscosity (at 50 °C); SB = FV−HPV: setback; P_t_: pasting temperature; BS: blank sample; 1_OS: octenyl succinate starch modified with 1% OSA; 3_OS: octenyl succinate starch modified with 3% OSA; 5_OS: octenyl succinate starch modified with 5% OSA; 7_OS: octenyl succinate starch modified with 7% OSA; BS+OA, 1_OS+OA, 3_OS+OA, 5_OS+OA, 7_OS+OA: corresponding starch samples mixed with oleic acid. Means within a column with the same letter are not significantly different (α < 0.05) by Fisher’s least significant difference test.

**Table 5:** Water permeability, water binding capacity, and water solubility of films prepared from octenyl succinate starches and their mixtures with oleic acid.

Film Component	Water Vapor Capacity	Water Binding Capacity (%)	Solubility in Water
	(10^−8^ g/s·m·kPa)		(%)
BS	6.97 ± 0.06 ^d^	9.02 ± 0.05 ^b^	38.3 ± 1.8 ^bc^
BS+OA	7.41 ± 0.23 ^b^	7.94 ± 1.12 ^e^	36.0 ± 2.4 ^cd^
1_OS	7.82 ± 0.29 ^ac^	7.58 ± 0.49 ^e^	40.9 ± 1.9 ^ab^
1_OS+OA	8.00 ± 0.06 ^a^	4.64 ± 0.60 ^d^	32.5 ± 0.3 ^e^
3_OS	7.95 ± 0.30 ^a^	7.44 ± 0.62 ^e^	40.3 ± 1.2 ^ab^
3_OS+OA	7.52 ± 0.26 ^bc^	6.95 ± 0.62 ^ce^	34.1 ± 2.0 ^de^
5_OS	7.92 ± 0.24 ^ab^	11.39 ± 1.33 ^a^	39.4 ± 2.4 ^b^
5_OS+OA	7.78 ± 0.10 ^ac^	4.67 ± 0.26 ^d^	28.9 ± 2.0 ^f^
7_OS	7.64 ± 0.28 ^bc^	Nd *	42.7 ± 2.2 ^a^
7_OS+OA	7.28 ± 0.26 ^d^	6.08 ± 0.22 ^c^	33.0 ± 2.4 ^de^

* Nd: above the limit of detection; BS: blank sample; 1_OS: octenyl succinate starch modified with 1% OSA; 3_OS: octenyl succinate starch modified with 3% OSA; 5_OS: octenyl succinate starch modified with 5% OSA; 7_OS: octenyl succinate starch modified with 7% OSA; BS+OA, 1_OS+OA, 3_OS+OA, 5_OS+OA, 7_OS+OA: corresponding starch samples mixed with oleic acid. Means within a column with the same letter are not significantly different (α < 0.05) by Fisher’s least significant difference test.

## Data Availability

Only the mean values and standard deviations are reported in the article. Raw data supporting these findings can be provided by the corresponding author upon request.

## Supplementary Materials

The following supporting information can be downloaded at: https://www.mdpi.com/article/10.3390/molecules30224411/s1, Figure S1: Normalized radar chart illustrating the relationships between: (A) complexing index and substitution with OS groups; (B) Peak viscosity of paste and water solubility at 80 °C. BS: blank sample; 1_OS: octenyl succinate starch modified with 1% OSA; 3_OS: octenyl succinate starch modified with 3% OSA; 5_OS: octenyl succinate starch modified with 5% OSA; 7_OS: octenyl succinate starch modified with 7% OSA.

## References

[1] Sweedman M.C., Tizzotti M.J., Schäfer C., Gilbert R.G. (2013). Structure and physicochemical properties of octenyl succinic anhydride modified starches: A review. Carbohydr. Polym..

[2] Królikowska K., Fortuna T., Pająk P., Witczak M. (2019). Impact of the degree of octenyl succinylation on metal ions complexation and functional properties of maize starch. Food Chem..

[3] Altuna L., Herrera M.L., Foresti M.L. (2018). Synthesis and characterization of octenyl succinic anhydride modified starches for food applications. A review of recent literature. Food Hydrocoll..

[4] Drusch S., Schwarz K. (2006). Microencapsulation properties of two different types of n-octenylsuccinate-derivatised starch. Eur. Food Res. Technol..

[5] Zhou J., Ren L., Tong J., Ma Y. (2009). Effect of surface esterification with octenyl succinic anhydride on hydrophilicity of corn starch films. J. Appl. Polym. Sci..

[6] Commision Regulation (EU) (2012). No 231/2012 9 March 2012 laying down specifications for food additives listed in Annexes II and III to Regulation (EC) No 1333/2008 of the European Parliament and of the Council. Off. J. Eur. Union.

[7] Magnusson E., Nilsson L. (2011). Interactions between hydrophobically modified starch and egg yolk proteins in solution and emulsions. Food Hydrocoll..

[8] Wu D., Lin Q., Singh H., Ye A. (2020). Complexation between whey protein and octenyl succinic anhydride (OSA)-modified starch: Formation and characteristics of soluble complexes. Food Res. Int..

[9] Wang S., Zheng M., Yu J., Wang S., Copeland L. (2017). Insights into the Formation and Structures of Starch-Protein-Lipid Complexes. J. Agric. Food Chem..

[10] Zhang G., Hamaker B.R. (2004). Starch-free fatty acid complexation in the presence of whey protein. Carbohydr. Polym..

[11] Lu X., Shi C., Zhu J., Li Y., Huang Q. (2019). Structure of starch-fatty acid complexes produced via hydrothermal treatment. Food Hydrocoll..

[12] Marinopoulou A., Papastergiadis E., Raphaelides S.N. (2016). An investigation into the structure, morphology and thermal properties of amylomaize starch-fatty acid complexes prepared at different temperatures. Food Res. Int..

[13] Copeland L., Blazek J., Salman H., Tang M.C. (2009). Form and functionality of starch. Food Hydrocoll..

[14] Handarini K., Sauman Hamdani J., Cahyana Y., Siti Setiasih I. (2020). Functional and pasting properties of a starch–lipid complex formed with gaseous ozone and palm oil. Int. J. Food Prop..

[15] Kang X., Yu B., Zhang H., Sui J., Guo L., El-Aty A.A., Cui B. (2021). The formation and in vitro enzymatic digestibility of starch-lipid complexes in steamed bread free from and supplemented with different fatty acids: Effect on textural and retrogradation properties during storage. Int. J. Biol. Macromol..

[16] Lalush I., Bar H., Zakaria I., Eichler S., Shimoni E. (2005). Utilization of amylose-lipid complexes as molecular nanocapsules for conjugated linoleic acid. Biomacromolecules.

[17] Wang S., Wang J., Yu J., Wang S. (2016). Effect of fatty acids on functional properties of normal wheat and waxy wheat starches: A structural basis. Food Chem..

[18] Królikowska K., Pietrzyk S., Łabanowska M., Kurdziel M., Pająk P. (2021). The influence of acid hydrolysis on physicochemical properties of starch-oleic acid mixtures and generation of radicals. Food Hydrocoll..

[19] Wang J., Su L., Wang S. (2010). Physicochemical properties of octenyl succinic anhydride-modified potato starch with different degrees of substitution. J. Sci. Food Agric..

[20] Choulis N.H. (2011). Miscellaneous Drugs, Materials, Medical Devices, and Techniques.

[21] Lopez-Huertas E. (2010). Health effects of oleic acid and long chain omega-3 fatty acids (EPA and DHA) enriched milks. A review of intervention studies. Pharmacol. Res..

[22] Królikowska K., Pietrzyk S., Pustkowiak H., Wolak K. (2022). The effect of cassava and wheat starches complexation with selected fatty acids on their functional properties. J. Food Sci. Technol..

[23] Pérez-Gallardo A., Bello-Pérez L.A., García-Almendárez B., Montejano-Gaitán G., Barbosa-Cánovas G., Regalado C. (2012). Effect of structural characteristics of modified waxy corn starches on rheological properties, film-forming solutions, and on water vapor permeability, solubility, and opacity of films. Starch/Staerke.

[24] Pająk P., Gałkowska D., Juszczak L., Khachatryan G. (2022). Octenyl succinylated potato starch-based film reinforced by honey-bee products: Structural and functional properties. Food Packag. Shelf Life.

[25] Pająk P., Socha R., Królikowska K., Grzyb J., Hetmańczyk J., Zachariasz P. (2025). Characterization of octenyl succinylated potato-starch based films enriched with extracts from various honey-bee products. Int. J. Biol. Macromol..

[26] Hassan B., Ali S., Chatha S., Hussain A.I., Zia K.M. (2018). Recent advances on polysaccharides, lipids and protein based edible films and coatings: A review. Int. J. Biol. Macromol..

[27] Ruan H., Chen Q., Fu M., Xu Q., He G. (2009). Preparation and properties of octenyl succinic anhydride modified potato starch. Food Chem..

[28] Kurdziel M., Królikowska K., Łabanowska M., Pietrzyk S., Michalec M. (2020). The effect of thermal and irradiation treatments on structural and physicochemical properties of octenyl succinate maize starches. Food Chem..

[29] Simsek S., Ovando-Martinez M., Marefati A., Sj M., Rayner M. (2015). Chemical composition, digestibility and emulsification properties of octenyl succinic esters of various starches. Food Res. Int..

[30] Wang J., Ren F., Yu J., Copeland L., Wang S. (2021). Octenyl Succinate Modification of Starch Enhances the Formation of Starch-Lipid Complexes. J. Agric. Food Chem..

[31] Wang S., Chao C., Cai J., Niu B., Copeland L., Wang S. (2020). Starch–lipid and starch–lipid–protein complexes: A comprehensive review. Compr. Rev. Food Sci. Food Saf..

[32] Eliasson A.-C. (1994). Interactions between starch and lipids studied by DSC. Thermochim. Acta.

[33] Nie H., Li C., Liu P., Lei C., Li J. (2019). Retrogradation, gel texture properties, intrinsic viscosity and degradation mechanism of potato starch paste under ultrasonic irradiation. Food Hydrocoll..

[34] Koyakumaru T., Nakano H. (2016). Thermal Characterization of the Gelatinization of Corn Starch Suspensions with Added Sodium Hydroxide or Urea as a Main Component of Corrugating Adhesives. J. Appl. Glycosci..

[35] Gelders G.G., Goesaert H., Delcour J.A. (2006). Amylose-lipid complexes as controlled lipid release agents during starch gelatinization and pasting. J. Agric. Food Chem..

[36] Chumsri P., Panpipat W., Cheong L.Z., Chaijan M. (2022). Formation of Intermediate Amylose Rice Starch–Lipid Complex Assisted by Ultrasonication. Foods.

[37] Ulbrich M., Flöter E. (2020). Modification of Starches with Different Amylose/Amylopectin-Ratios Using the Dual Approach with Hydroxypropylation and Subsequent Acid-Thinning—Impacts on Morphological and Molecular Characteristics. Starch/Staerke.

[38] Chung H.J., Lee S.E., Han J.A., Lim S.T. (2010). Physical properties of dry-heated octenyl succinylated waxy corn starches and its application in fat-reduced muffin. J. Cereal Sci..

[39] Liang X., King J.M., Shih F.F. (2002). Pasting property differences of commercial and isolated rice starch with added lipids and β-cyclodextrin. Cereal Chem..

[40] Zhao S., Tian G., Zhao C., Lu C., Bao Y., Liu X., Zheng J. (2018). Emulsifying stability properties of octenyl succinic anhydride (OSA) modified waxy starches with different molecular structures. Food Hydrocoll..

[41] Krstonošić V., Dokić L., Milanović J. (2011). Micellar properties of OSA starch and interaction with xanthan gum in aqueous solution. Food Hydrocoll..

[42] Królikowska K., Fortuna T., Pietrzyk S., Gryszkin A. (2017). Effect of modification of octenyl succinate starch with mineral elements on the stability and rheological properties of oil-in-water emulsions. Food Hydrocoll..

[43] Naseri A., Shekarchizadeh H., Kadivar M. (2019). Octenylsuccination of sago starch and investigation of the effect of calcium chloride and ferulic acid on physicochemical and functional properties of the modified starch film. J. Food Process Preserv..

[44] Karnwal A., Rauf A., Jassim A.Y., Selvaraj M., Al-Tawaha A.R.M.S., Kashyap P., Kumar D., Malik T. (2025). Advanced starch-based films for food packaging: Innovations in sustainability and functional properties. Food Chem X..

[45] Osés J., Fernández-Pan I., Mendoza M., Maté J.I. (2009). Stability of the mechanical properties of edible films based on whey protein isolate during storage at different relative humidity. Food Hydrocoll..

[46] Wang L., Wang W., Wang Y., Xiong G., Mei X., Wu W., Ding A., Li X., Qiao Y., Liao L. (2018). Effects of fatty acid chain length on properties of potato starch–fatty acid complexes under partially gelatinization. Int. J. Food Prop..

[47] (2001). Starches, Native or Modified—Determination of Total Lipid Content.

[48] Vilaplana F., Gilbert R.G. (2010). Characterization of branched polysaccharides using multiple-detection size separation techniques. J. Sep. Sci..

[49] Coseri S., Bercea M., Harabagiu V., Budtova T. (2016). Oxidation vs. degradation in polysaccharides: Pullulan—A case study. Eur. Polym. J..

[50] Li G., Wang S., Zhu F. (2016). Physicochemical properties of quinoa starch. Carbohydr. Polym..

[51] Lima E.R.A., De Melo B.M., Baptista L.T., Paredes M.L.L. (2013). Specific ion effects on the interfacial tension of water/hydrocarbon systems. Braz. J. Chem. Eng..

[52] Ryu S.Y., Rhim J.W., Roh H.J., Kim S.S. (2002). Preparation and physical properties of zein-coated high-amylose corn starch film. Lwt.

[53] Šuput D., Lazić V., Pezo L., Markov S., Vaštag Ž., Popović L., Radulović A., Ostojic S., Zlatanovic S., Popović S. (2016). Characterization of starch edible films with different essential oils addition. Pol. J. Food Nutr. Sci..

[54] Kumaran M.K. (1998). Interlaboratory Comparison of the ASTM Standard Test Methods for Water Vapor Transmission of Materials (E 96-95). J. Test. Eval..

