# Supplementary material for: Comprehensive Evaluation of OS Starch–Oleic Acid Mixtures: From Functional Properties to Their Application in Films with Improved Water Resistance
Source: Molecules. 2025 Nov 14;30(22):4411. doi: 10.3390/molecules30224411 (PMC12655613; doi:10.3390/molecules30224411)
Supplement: Supplementary file 1 [file molecules-30-04411-s001.zip › molecules-3954091-supplementary.pdf]

Supplementary Material

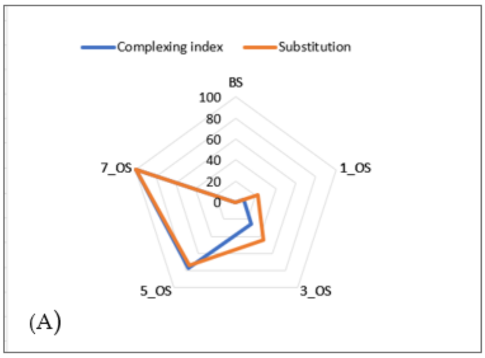

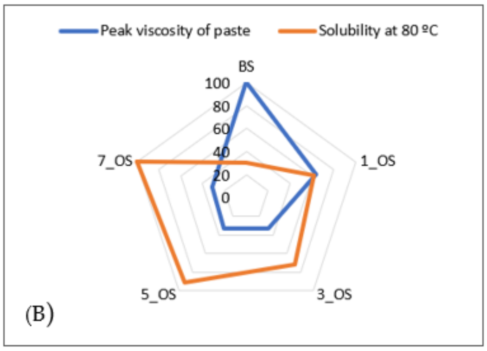

Supplementary Figure S1. Normalized radar chart illustrating the relationships between: (A) complexing index and substitution with OS groups; (B) Peak viscosity of paste and water solubility at 80 °C. BS: blank sample; 1_OS: octenyl succinate starch modified with 1% OSA; 3_OS: octenyl succinate starch modified with 3% OSA; 5_OS: octenyl succinate starch modified with 5% OSA; 7_OS: octenyl succinate starch modified with 7% OSA.
